# NIR pH-Responsive PEGylated PLGA Nanoparticles as Effective Phototoxic Agents in Resistant PDAC Cells

**DOI:** 10.3390/polym17081101

**Published:** 2025-04-18

**Authors:** Degnet Melese Dereje, Francesca Bianco, Carlotta Pontremoli, Alessandra Fiorio Pla, Nadia Barbero

**Affiliations:** 1 NIS Interdepartmental and INSTM Reference Centre, Department of Chemistry, University of Torino, Via G. Quarello 15A, 10135 Torino, Italy; degnetmelese.dereje@unipd.it (D.M.D.); nadia.barbero@unito.it (N.B.); 2 Bahir Dar Institute of Technology, Department of Chemical Engineering, Bahir Dar University, Polypeda 01, Bahir Dar 0026, Ethiopia; 3 Department of Life Sciences and Systems Biology, University of Torino, Via Accademia Albertina 13, 10123 Turin, Italy; f.bianco@unito.it (F.B.); alessandra.fiorio@unito.it (A.F.P.); 4 Department of Chemistry, Biology and Biotechnology, University of Perugia, Via dell’ Elce di Sotto 8, 06123 Perugia, Italy; 5 Institute of Science and Technology for Ceramics (ISSMC-CNR), Via Granarolo, 64, 48018 Faenza, Italy

**Keywords:** photodynamic therapy, heptamethine-cyanine dyes, PEGylated PLGA nanoparticles, resistant PDAC cells

## Abstract

Pancreatic ductal adenocarcinoma (PDAC) is one of the deadliest cancers worldwide due to its resistance to conventional therapies that is attributed to its dense and acidic tumor microenvironment. Chemotherapy based on gemcitabine usually lacks efficacy due to poor drug penetration and the metabolic characteristics of the cells adapted to grow at a more acidic pH_e_, thus presenting a more aggressive phenotype. In this context, photodynamic therapy (PDT) offers a promising alternative since it generally does not suffer from the same patterns of cross-resistance observed with chemotherapy drugs. In the present work, a novel bromine-substituted heptamethine-cyanine dye (BrCY7) was synthesized, loaded into PEG-PLGA NPs, and tested on the pancreatic ductal adenocarcinoma cell line cultured under physiological (PANC-1 CT) and acidic (PANC-1 pH selected) conditions, which promotes the selection of a more aggressive phenotype. The cytotoxicity of BrCY7-PEG-PLGA is dose-dependent, with an IC_50_ of 2.15 µM in PANC-1 CT and 2.87 µM in PANC-1 pH selected. Notably, BrCY7-PEG-PLGA demonstrated a phototoxic effect against PANC-1 pH selected cells but not on PANC-1 CT, which makes these findings particularly relevant since PANC-1 pH selected cells are more resistant to gemcitabine as compared with PANC-1 CT cells.

## 1. Introduction

Pancreatic ductal adenocarcinoma (PDAC) is one of the leading causes of mortality all over the world as a consequence of its strong resistance to medical therapies, correlated with its microenvironment. In fact, the tumor microenvironment (TME) of PDAC is characterized by a dense desmoplastic stroma, thanks to the presence of non-tumoral cells that massively secrete extracellular matrix in the TME, such as cancer associated fibroblasts (CAFs) and pancreatic stromal cells (PSCs) [1]. This stiffer and complex environment makes it difficult for the blood vessels to reach the internal tumor core and for the lymphatic vessels to remove excess fluids. The direct consequence is a reduction in oxygen and nutrient diffusion into the tumor mass, making the tumor core strongly hypoxic and acidic [2,3]. This induces dysregulation in a series of intracellular pathways, which phenotypically results in the epithelial-to-mesenchymal (EMT) transition of the cancer cells, increasing their ability to migrate and invade [4]. In fact, we recently demonstrated that acidic pH_e_ selects for a more aggressive PDAC phenotype, with an increased ability to migrate and invade [5]. To date, the first-line therapeutic strategy for the treatment of PDAC remains surgery, only available for patients with localized lesions, which represents only a minority of the cases. Indeed, the majority of patients are diagnosed with metastatic diseases and so are ineligible for surgery. Broad spectrum chemotherapy is, therefore, offered as treatment with a first-line setting consisting of FOLFIRINOX (5-fluorouracil, leucovorin, irinotecan, and oxaliplatin) or gemcitabine-based combination therapies [6,7]. However, these treatments have resulted in real but modest survival improvement due to the resistance of PDAC cells to chemotherapy [8] caused by the low diffusion capability of the drugs and their metabolic characteristics. Based on these considerations, it is, therefore, clear that the development of a new strategy for PDAC treatment is crucial.

Among the new rising therapies, the use of photodynamic therapy (PDT) seems to be promising. PDT is a medical treatment that harnesses the power of light and photosensitive drugs, used to treat several diseases, including PDAC cancer [6]. This innovative approach involves the administration of a photosensitizing agent (PS), which selectively accumulates in target tissues or cells. When activated by light of a specific wavelength, the photosensitizer generates singlet oxygen ^1^O_2_ and/or reactive oxygen species (ROS), leading to localized cell death or destruction of targeted structures. Compared to conventional treatments, such as surgery, chemotherapy, and radiation therapy, PDT offers several advantages thanks to the non-invasive nature of the treatment [9,10]: the high selectivity in tumor destruction [11], which allows for a reduction in damage to surrounding healthy tissues, and the possibility of repeated treatments without exceeding dose limits or causing systemic complications, thanks to the short duration of the ^1^O_2_ and ROS generation in localized areas [12].

The PDT response is strictly dependent on the intrinsic nature and the photochemical properties of the PS. In particular, near-infrared (NIR) photosensitizers are extremely useful in biomedical applications, thanks to the deeper penetration of the NIR light into tissues, allowing for the treatment of tumors or diseased tissues located at greater depths, as well as the lower scattering compared to visible light [13]. Among them, cyanine dyes (CYs) are known for their outstanding photophysical and photochemical properties, such as sharp and intense absorption bands and narrow emission bands with high extinction coefficients in the red and NIR, high fluorescence quantum yield and low dark toxicity and side effects, and strong fluorescent emission in organic solvents [14,15,16,17]. Compared to pentamethine counterparts, the extended conjugated system in heptamethine CY dyes enables them to absorb and emit light at longer wavelengths, typically ranging from 700 to 900 nanometers, which makes them the perfect candidates for the treatment of tumors with dense stroma, such as PDAC, thanks to a better light penetration. However, they suffer from limited stability, since heptamethine CY can be prone to photobleaching, especially under prolonged exposure to light. This can lead to a decrease in absorbance and fluorescence intensity over time, affecting the ROS production and their application in PDT. Moreover, the limited solubility of this class of dyes in aqueous media can lead to aggregation or precipitation, resulting in compromised photochemical properties, with a consequent altered PDT effect. To address these limitations, a possible strategy is represented by their loading into nanoparticle-based delivery systems. Nanoparticles provide a versatile platform for encapsulating or conjugating CY dyes, enhancing their solubility, stability, and bioavailability [18]. In this context, heptamethine-cyanine dyes were incorporated into SiO_2_ [19] and Fe_2_O_3_ [20] NPs to address limitations in physiological conditions, showing higher stability in water and improved singlet oxygen production capacity compared to the free dye. Zhang showed how the photothermal and photodynamic activities of ICG increased after encapsulation into NPs [21].

In the field of biodegradable NPs, considering the well-established clinical safety profile and advantageous physicochemical properties, poly lactic-co-glycolic acid (PLGA) NPs can be considered a good option to load cyanine dyes for application in PDT [22,23,24,25,26,27]. However, challenges such as rapid clearance from systemic circulation, ineffective targeting leading to side effects, and limited cellular uptake of negatively charged PLGA NPs hinder their effectiveness in crossing narrow barriers [28,29]. To overcome these challenges and achieve prolonged systemic circulation, improved aqueous solubility, suppressed opsonization, and reduced aggregate formation, we explored the functionalization of a highly biocompatible nonionic hydrophilic polyether, in particular, PEG (polyethylene glycol), through conjugation with PLGA NPs [28,30,31].

In this contribution, we successfully synthesized and fully characterized a novel bromine-substituted, indolenine-based heptamethine-cyanine dye (BrCY7), showing a good ROS production, thanks to the presence of bromine atoms, already proven to induce the singlet-to-triplet state intersystem crossing [15] and absorption in the near-infrared (NIR) region. Due to the photobleaching and tendency to self-aggregate in biological media and complete insolubility in physiological conditions, BrCY7 was incorporated into pegylated PLGA NPs and then tested against pancreatic ductal adenocarcinoma cell line PANC-1, equilibrated at physiological pH_e_ (PANC-1 CT), and cultured for one month at pH_e_6.6 (PANC-1 pH selected), which has been demonstrated by our laboratory to be a promising model for mimicking the behavior of the acidic core of PDAC [5].

## 2. Materials and Methods

### 2.1. Chemicals and Reagents

Chemicals were purchased from VWR, Merck, or TCI and were used without any further purification, and all organic solvents were analytical grade. Resomer^®^ RG 653 H (65:35) with Mw 24,000–38,000 Da, Pluronic F-127 with Mw ~12,600 g/mol, and Hydroxy Polyethylene Glycol Amine with Mw 3000 Da were purchased from Merck.

5-bromo-2,3,3-trimethyl-3H-indole (**1**) [32] and N-((E)-((E)-2-chloro-3-((phenylamino)methylene)cyclohex-1-en-1-yl)methylene)benzenaminium chloride (**3**) [33] were prepared as previously described.

Microwave reactions were carried out in single-mode Biotage Initiator+.

High-resolution mass spectrometry (HRMS) analyses were carried out using an Orbitrap IQ-X (Thermo Fisher Scientific, Waltham, MA, USA) equipped with a heated electrospray ionization (HESI) source. The ion source was operated in positive ion mode with these settings: spray voltage of 3.3 kV (+), ion transfer tube temperature of 290 °C, and vaporizer temperature of 300 °C. Nitrogen served as both sheath gas and auxiliary gas, with flow rates set at 40 and 10 arbitrary units (a.u.), respectively. HRMS spectrum was acquired in the *m*/*z* range of 100–1000 at a target resolution of 240,000 (at 200 *m*/*z* FWHM). The observed mass error was below 1 ppm. Data analysis was performed using FreeStyle software (v1.8 SP2, Thermo Fisher Scientific, Waltham, MA, USA).

^1^H NMR (600 MHz) and ^13^C NMR (151 MHz) spectra were recorded at 25 °C on a Jeol ECZR NMR (Jeol, Milan, Italy) in CDCl_3_ and DMSO-*d*_6_; the deuterated solvent was used for calibration.

### 2.2. Synthesis and Characterization of Bromine-Substituted Cyanine Dye (BrCY7)

#### 2.2.1. Synthesis of BrCY7 Dye

*Synthesis of Bromo-1-butyl-2,3,3-trimethyl-3H-indol-1-ium iodide* (**2**). 5-bromo-2,3,3-trimethyl-3H-indole (**1**) (500 mg, 2.1 mmol), 1-iodobutane (0.7 mL, 6.3 mmol), and acetonitrile (2 mL) were weighed in a reaction vial, successively sealed with a crimp cap, and heated in a microwave system using the following parameters: temperature 155 °C and time 30 min. The crude solid was obtained after evaporation of the solvent under vacuum and washed three times with diethyl ether (3 × 50 mL). After filtration, the compound was obtained as a brownish solid (603 mg, 68% yield).

^1^H NMR (600 MHz, DMSO-d6): δ = 8.20 (s, 1H), 7.97 (d, *J* = 8.0 Hz, 1H), 7.84 (d, *J* = 8.0 Hz, 1H), 4.45 (mt, *J* = 7.0 Hz, 2H), 2.85 (s, 3H), 1.87–1.72 (m, 2H), 1.55 (s, 6H), 1.47–1.36 (m, 2H), 0.92 (t, *J* = 7.5 Hz, 3H) [15,32].

Synthesis of BrCY7 (5-bromo-2-((E)-2-((E)-3-(2-((Z)-5-bromo-1-butyl-3,3-dimethylindolin-2-ylidene)ethylidene)-2-chlorocyclohex-1-en-1-yl)vinyl)-1-butyl-3,3-dimethyl-3H-indol-1-ium iodide): Compounds 2 (295 mg, 0.7 mmol), N-((E)-((E)-2-chloro-3-((phenylamino)methylene)cyclohex-1-en-1-yl)methylene)benzenaminium chloride (**3**) (100 mg, 0.28 mmol), anhydrous potassium acetate (68 mg, 0.7 mmol), and absolute ethanol (2 mL) were weighed in a microwave vial, and the following parameters were set: temperature 120 °C and time 15 min. The obtained suspension was poured dropwise in diethyl ether (100 mL) to promote the precipitation of a brown solid and finally washed with diethyl ether (3 × 50 mL) and filtered. The filtered brown solid was dissolved in DCM (20 mL), and the unreacted potassium acetate crystals were removed by filtration. The final gold/green crystals (120 mg, 56% yield) were obtained by evaporating the solvent under vacuum and after crystallization in ACN.

^1^H NMR (Figure S1, 600 MHz, CDCl_3_) δ 8.30 (d, *J* = 14.1 Hz, 2H), 7.51 (dd, *J* = 8.4, 1.9 Hz, 2H), 7.46 (d, *J* = 1.8 Hz, 2H), 7.05 (d, *J* = 8.4 Hz, 2H), 6.27 (d, *J* = 14.1 Hz, 2H), 4.22 (t, *J* = 7.4 Hz, 4H), 2.76 (t, *J* = 6.2 Hz, 4H), 1.98 (t, *J* = 6.1 Hz, 2H), 1.81 (ddt, *J* = 9.6, 7.7, 3.6 Hz, 4H), 1.71 (s, 12H), 1.52–1.44 (m, 4H), 1.00 (t, *J* = 7.4 Hz, 6H).

^13^C NMR (Figure S2, 151 MHz, CDCl_3_) δ 171.74, 144.29, 143.13, 141.62, 131.92, 128.61, 125.78, 118.45, 112.50, 102.27, 77.37, 77.16, 76.95, 49.43, 45.38, 29.85, 29.62, 28.25, 27.02, 20.50, 14.08.

HRMS (ESI) *m*/*z*, Figure S3: [M-I]^+^ calcd for [C_38_H_46_Br_2_ClN_2_]^+^ 725.1691 and 723.1711, found 725.1702 and 723.1716.

UV–Vis (DMSO): *λ*_max_ (*ε*) = 795 nm (119,001 M^−1^cm^−1^).

#### 2.2.2. Spectroscopic Characterization of BrCY7 Dye

*UV*–*Vis spectroscopy.* UV–Vis spectra were acquired using a Cary 300 Bio spectrophotometer (Varian, Santa Clara, CA, USA) by solubilizing the powder in DMSO and PBS. The stock solution was analyzed at room temperature after proper dilutions in the range of 300–900 nm.

*Determination of molar extinction coefficient.* To determine the molar extinction coefficient (ε), a 0.5 mM stock solution of BrCY7 in DMSO was initially prepared. Serial dilutions were then made by withdrawing appropriate aliquots to obtain solutions with final concentrations of 2.0, 3.0, 4.0, 5.0, and 6.0 μM. UV–Vis absorption spectra were recorded in the 500–900 nm range using quartz cuvettes with a 1 cm path length. The absorbance values at the absorption maximum (λ_max_) were plotted against the corresponding concentrations. A linear regression was used to extract the molar extinction coefficient, calculated as the slope of the resulting line. Each measurement was performed in duplicate, and data were accepted only when the variation in log ε between replicates did not exceed 0.02 from the mean.

*Fluorescence spectroscopy.* Fluorescence spectra were acquired using a HORIBA Jobin Yvon Fluorolog-3 spectrofluorometer (HORIBA, Kyoto, Japan) within the 750–900 nm range. Excitation was performed at the cyanine shoulder identified from the corresponding UV–Vis spectra. Both the excitation and emission slit widths were set to 5 nm. To minimize aggregation effects, measurements were conducted on diluted solutions with absorbance values approximately ≤0.1.

### 2.3. Activation and PEGylation of PLGA Resomer

*Activation of PLGA*. Before PEGylation, the COOH group of Resomer^®^ RG 653 H (65:35) was activated with N,N′-Dicyclohexylcarbodiimide (DCC) and N-Hydroxysuccinimide (NHS) coupling agent following a protocol reported in the literature [34]. Specifically, Resomer^®^ RG 653 H (65:35) (3720 mg; 0.12 mmol), DCC (49.5 mg; 0.24 mmol), and NHS (27.6 mg; 0.24 mmol) coupling agents (molar ratio 1:2:2) were solubilized in anhydrous DCM (4 mL) and stirred overnight under nitrogen atmosphere. The resulting solution was subsequently filtered using a nylon syringe filter with 0.45 µm porosity to remove the DCC by-product. Then, the filtered solution was precipitated in ice ether, followed by repeated washing with diethyl ether and methanol to remove unreacted NHS. Finally, the activated PLGA was dried under vacuum.

*PEGylation of PLGA*. Activated PLGA (1.288 mg; 0.041 mmol) was dissolved in DCM and dropped to a solution of hydroxyl-PEG-amine (125 mg; 0.041 mmol) previously dissolved in DCM with the ratio of PLGA and PEG equal to 1:1. The reaction was stirred for 6 h in nitrogen atmosphere, and the product was precipitated with cold methanol. The solution was then washed with methanol, filtered using nylon syringe filter with a 0.45 µm porosity, and centrifuged at 4000 rpm, 4 °C for 30 min to separate the precipitate from the solvent (methanol) and dried in the vacuum oven. The obtained powder is thereafter named as PEG-PLGA.

### 2.4. Preparation of BrCY7-Loaded PEGylated PLGA Nanoparticles

To improve its stability and solubility in biological environments, BrCY7 was incorporated into the prepared PEG-PLGA NPs. Based on previous experiments reported by the authors, an optimized modified single-emulsion method was employed to prepare both empty and BrCY7-loaded PEG-PLGA nanoparticles [35]. Briefly, to prepare empty PEG-PLGA NPs, the process involves the preparation of an organic phase, where 70 mg of PEG-PLGA is dissolved in DCM. The aqueous phase is created by dissolving Pluronic F-127 in 10 mL of water. Subsequently, the organic phase is combined with the aqueous phase, and immediate sonication is carried out in pulse mode, with a 50% sonication cycle and an output power of 40%, lasting for 30 s by using a sonication probe (Sonics Vibra Cell VC375 Ultrasonic Processor, Sonics and Materials, Newtown, CT, USA).

Concerning dye-loaded PEG-PLGA, the procedure remained unchanged, except for dissolving 1.5 mg of BrCY7 in 3 mL of DCM before the addition to the aqueous phase. During the sonication, the container was maintained in an ice bath to reduce the temperature increase. Subsequently, the organic solvent was evaporated under stirring at room temperature in a fume hood for 4 h.

The obtained suspension was then centrifuged (using a JOUAN MR23i Benchtop High Speed Centrifuge, Thermo Scientific MR23i, Thermo Fisher Scientific, Waltham, MA, USA) at 10,000 rpm for 30 min to collect NPs, followed by three washing steps with deionized water. Finally, the nanoparticles were resuspended in water, and the powder was obtained after a freeze-drying process. The obtained powders are thereafter named as PEG-PLGA NPs and BrCY7-PEG-PLGA.

### 2.5. Yield and Encapsulation Efficiency (EE%)

The amount of BrCY7 dye incorporated into PEG-PLGA NPs was calculated by a direct method using UV–Vis spectroscopy. A calibration curve of free BrCY7 in DMSO (at the absorption wavelength of 795 nm) was prepared as reported in 2.2.2 section. Then, 2 mg of freeze-dried BrCY7-PEG-PLGA was dissolved in DMSO and sonicated for 5 min to force the release of the dye in the solution. By using the Lamber–Beer equation (molar extinction coefficient (ε) in DMSO of BrCY7 = 119,001 M^−1^cm^−1^), the concentration of the released dye was calculated. The final entrapment efficiency and drug loading content were calculated using the following standard Equations (1) and (2) [35]:

(1) $$Encapsulation\;efficacy\;EE\;(\%)=\frac{mg\;of\;released\;BrCY7}{mg\;of\;initial\;weighted\;BrCY7}\cdot 100$$

(2) $$Loading\;content\;LC\;(\%)=\frac{Mass\;of\;incorporated\;BrCY7}{Mass\;of\;BrCY7-PEG-PLGA}\cdot 100$$

### 2.6. Characterization of BrCY7-Loaded PEG-PLGA Nanoparticles

*Nanoparticle tracking analysis (NTA).* Nanoparticle tracking analysis (NTA) was employed to evaluate the mean particle diameter (MPD), SPAN (an indicator of particle size distribution), and zeta potential (ζ-potential) of both empty and drug-loaded nanoparticles. Analyses were performed using a ZetaView^®^ PMX-120 mono-laser system (YG-488, Particle Matrix, Inning am Ammersee, Germany), operating with software version 8.05.14_SP7. Measurements were conducted at 25 °C with a 488 nm laser under the following conditions: mobility profile assessment across 11 positions, with Max Area set to 10,000, Min Area set to 10, and Min Brightness set to 25. Prior to sample analysis, the instrument was calibrated using a 100 nm polystyrene standard suspension (1 mL). Subsequently, 1 mL of each sample was introduced into the sample port. Zeta potential was determined at two fixed positions during the measurement.

*Field Emission Electron Microscopy (FE-SEM).* The morphology and size of both empty and dye-loaded nanoparticles were examined using field emission scanning electron microscopy (FE-SEM) with a TESCAN S9000G instrument (TESCAN, Brno, Czechia) equipped with a Schottky emitter and a resolution of 0.7 nm in in-beam SE mode at 15 keV. For analysis, the dry nanoparticle powders were directly deposited onto conductive carbon tape and subsequently coated with a 7 nm chromium (Cr) layer to enhance conductivity.

### 2.7. Measurement of Reactive Oxygen Species (ROS) and Evaluation of the Photodegradation

The generation of reactive oxygen species (ROS) by BrCY7-PEG-PLGA was assessed using 1,3-diphenylisobenzofuran (DPBF) as a ROS-sensitive probe following a method adapted from previously published protocols [36]. DPBF stock solutions were prepared in DMSO, whereas BrCY7-PEG-PLGA stock was dissolved directly in phosphate-buffered saline (PBS, 2 mM, pH 7.4). Both solutions were subsequently diluted in PBS to reach the desired final concentration, with DPBF at 25 μM. Samples were placed in quartz cuvettes (1 cm path length) and exposed to light in an aerated Solarbox 3000e photoreactor (250 W xenon lamp, CO.FO.ME.GRA, Milan, Italy). To prevent DPBF photodegradation, illumination was carried out using a 515 nm cut-off optical filter. Absorbance spectra were recorded at defined time intervals, monitoring the decrease in the characteristic DPBF peak at 415 nm over time as an indicator of ROS production. Due to the instability and solubility issues of free dye in PBS, the ROS production of free BrCY7 was not evaluated. Moreover, to assess the effectiveness of the nanoparticle in protecting the molecule from photodegradation after incorporation, the photodegradation test was evaluated by irradiating BrCY7-PEG-PLGA prepared in PBS with the same parameters used to assess ROS generation. Absorption spectra were recorded at different time points, and absorbance at 812 nm was plotted as a function of irradiation time.

### 2.8. Biological Assessment

#### 2.8.1. Cell Culture

Human pancreatic tumor cell lines (PANC-1) were kindly provided by the Institute for Experimental Cancer Research, Christian-Albrecht-University (CAU) of Kiel, Germany. We maintained these cells in both physiological (PANC-1 CT) and acidic (PANC-1 pH selected) media as previously described [5].

#### 2.8.2. Cytotoxicity and Photoactivity

To investigate the cytocompatibility of the empty PEG-PLGA NPs and BrCY7-PEG-PLGA, PANC-1 cells, both CT and pH selected, were seeded in 96-well plates at a density of 0.5 × 10^4^ cells/well. Once attached, PANC-1 cells, both control (CT) and pH selected, were treated with different concentrations of PEG-PLGA NPs [500 µg/mL, 750 µg/mL, 1 mg/mL, and 1.250 mg/mL] and BrCY7-PEG-PLGA [2 µM, 3 µM, 4 µM, and 5 µM] prepared directly in complete medium, and cell viability was assessed 48, 72, and 96 h after treatment. Cell viability was assessed using the CellTiter 96^®^ Aqueous Non-Radioactive cell proliferation assay (Promega, Madison, WI, USA). The reagent was added to each well and incubated for 2 h at 37 °C in the dark. Following incubation, absorbance at 490 nm was measured using a FilterMaxF5 Multi-Mode Microplate Reader (Molecular Devices, San Jose, MO, USA). Each condition was tested in four technical replicates, and a minimum of three independent experiments were conducted. Absorbance readings were normalized against untreated control cells at 48 h (CTR) and interpreted as an indicator of viable cell number.

To evaluate the photodynamic effect of BrCY7-PEG-PLGA, PANC-1 cells, both CT and pH selected, were seeded in 96-well plates at a density of 0.5 × 10^4^ cells/well. Once attached, the cells were treated with 2 µM BrCY7 loaded in 500 µg/mL PEG-PLGA and incubated O/N at 37 °C and 5% CO_2_. The day after, the cells were irradiated for 15 min with a RED-LED array (96 LEDs in a 12 × 8 arrangement, excitation wavelength: 770 nm, Fluence 7.2 J/cm^2^, Irradiance 8 mW/cm^2^, in line with previous literature [37,38]) specifically designed and produced by Cicci Research s.r.l (Grosseto, Italy). Cell viability was assessed 24, 48, and 72 h after irradiation (48, 72, and 96 h after BrCY7-PEG-PLGA treatment) as described above. Four technical replicates for each condition and at least three independent experiments were performed, and absorbance values were normalized to the 24 h untreated cells (CTR) and analyzed as proportional to the number of viable cells.

Gemcitabine (Selleckchem Cat#S1149) cytotoxicity was evaluated on both PANC-1 CT and pH selected cells to test their different resistance to chemotherapy and was also used as the gold standard to compare our BrCY7-PEG-PLGA. To test whether pH selected PANC-1 cells are more resistant to gemcitabine compared to PANC-1 CT cells, in accordance with previous literature [8], both cell lines were plated (0.5 × 10^4^ cells/well) in a 96-well plate. Once attached, these were treated with gemcitabine [5 µM, 10 µM, 50 µM], and the viability was assessed at 72 h post-treatment using the MTS assay as described above. Each condition was tested in four technical replicates, and a minimum of three independent experiments were conducted. Absorbance readings were normalized against untreated control cells (CTR) and interpreted as an indicator of viable cell number.

#### 2.8.3. IC_50_ Determination

The IC_50_ (half-maximal inhibitory concentration) was assessed at 96 h post-treatment. Both PANC-1 CT and PANC-1 pH selected cells were seeded in 96-well plates (0.5 × 10^4^ cells/well). Once the cells were attached, they were treated with BrCY7-PEG-PLGA [0.1 µM, 0.5 µM, 1 µM, 2 µM, 3 µM, 4 µM, and 5 µM]. At 96 h post-treatment, cell viability was assessed as described above, normalizing each condition on the untreated cells (CTR). The results are expressed in % of cell viability and used to calculate the IC_50_ from the curves by interpolation using GraphPad Prism 6.0 software. At least three biological replicates with four technical replicates were performed for each condition.

### 2.9. Statistical Analysis

Data are expressed as means ± SEM (standard error mean) and refer to at least three independent experiments. Statistical analyses were performed using Graph-Pad Prism 6.0 software (La Jolla, CA, USA). Statistical significance among populations was determined by analysis of variance (RM one-way ANOVA without Geisser–Greenhouse correction or Friedman according to data distribution), followed by Dunnett’s or Dunn’s multiple comparisons post hoc test to compare more than two conditions in the cytotoxicity and photo-toxicity assays. Differences with *p*-values < 0.05 were considered statistically significant: *: *p*-value < 0.05, **: *p*-value < 0.01, ***: *p*-value < 0.001, ****: *p*-value < 0.0001.

## 3. Results and Discussion

### 3.1. Synthesis and Physicochemical Characterization of BrCY7

**Scheme 1:**
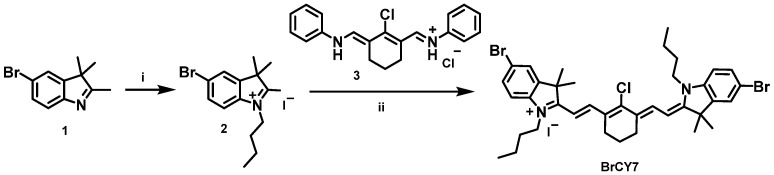
Synthesis of BrCY7. (i) 1-iodobutane and acetonitrile, microwave heating, 155 °C for 30 min; (ii) anhydrous potassium acetate and absolute ethanol, microwave heating, 120 °C for 15 min.

Cyanine dyes have been explored as potential PS for PDT since their photochemical properties are well suited for this application. To move towards the near-infrared (NIR) region, we successfully synthesized a bromine-substituted heptamethine indolenine cyanine dye (BrCY7) with an absorption peak at 795 nm. This molecule was rationalized to bear two bromine atoms, strategically introduced to enhance intersystem crossing from the singlet to the triplet state via the well-documented heavy atom effect [15]. The synthetic route to BrCY7 (illustrated in Scheme 1) began with the quaternization of a bromoindolenine precursor (**1**), prepared according to established literature protocols [32,36], yielding intermediate compound **2**.

**Figure 1:**
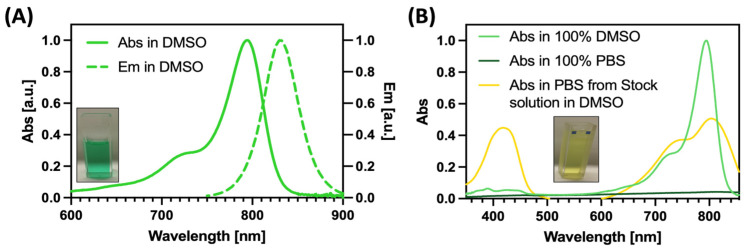
Spectroscopic characteristics of BrCY7 in DMSO (Abs: absorption; Em: emission) (**A**) and comparison of absorbance behavior in DMSO and PBS (**B**).

The reaction, performed under microwave irradiation, led to an increased acidity of the methyl group, thereby facilitating its subsequent condensation with the polymethine bridge (**3**) stabilized by a classical cyclohexene unit bearing a chlorine substituent. The final compound was obtained through a one-step microwave reaction. The presence of the central chlorine atom, known for its electron-withdrawing effect, further delocalized electron density along the polymethine chain, contributing to a bathochromic shift of the absorption into the NIR region. As illustrated in Figure 1, BrCY7 exhibits a strong absorption peak at approximately 795 nm in DMSO, with a very high molar extinction coefficient (119,001 M^−1^cm^−1^, see Figure S4).

**Figure 2:**
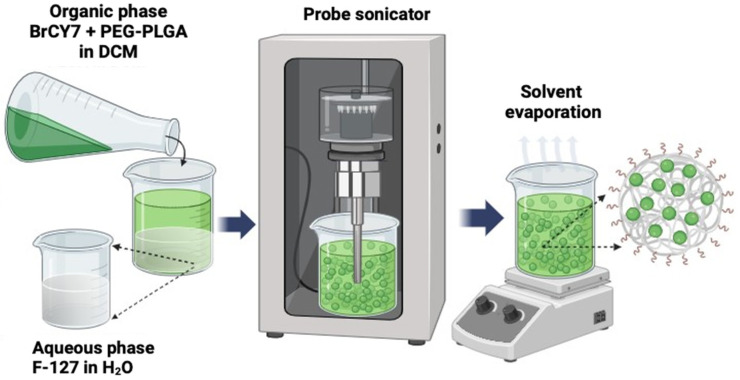
Synthetic workflow for the PEG-PLGA and BrCY7-PEG-PLGA nanoparticle preparation via the single-emulsion method.

The UV–Vis absorption spectrum of BrCY7 shows a sharp and well-defined band in the near-infrared (NIR) region, a key feature for effective photodynamic therapy (PDT). Additionally, a characteristic hypsochromic shoulder appears around 730 nm, a common spectral trait of cyanine dyes. Upon excitation at this shoulder, BrCY7 exhibits strong fluorescence, with an emission maximum centered at 830 nm (Figure 1A). On the other hand, when BrCY7 is dissolved in 100% PBS (Figure 1B, dark green line), the absorption is completely quenched due to the formation of aggregates. Moreover, the absorption spectrum of the cyanine dye pre-solubilized in DMSO and then diluted in PBS was evaluated. As shown in Figure 1B (yellow line), along with the formation of aggregates (evident from the change in the shape of the main absorption peak), the molecule undergoes degradation, leading to the breakdown of its structure and the appearance of an absorption peak between 350 and 450 nm. In fact, the solution rapidly turns from green to yellow as depicted in the Figure 1 inset. Based on these considerations, in order to preserve the photochemical properties essential for the PDT activity, the molecule was incorporated into PEG-PLGA NPs. Various methods for preparing both empty and loaded PEGylated PLGA NPs have been documented in the literature [34,39,40], with single emulsion (Figure 2) identified as the optimal formulation method for encapsulating polymethine dyes, as detailed in our recently published report [35].

As reported in the following paragraphs, both empty PEGylated PLGA (PEG-PLGA NPs) and BrCY7-PEG-PLGA were then fully characterized in terms of morphology, spectroscopic and physicochemical characteristics, as well as the ability to produce ROS before being tested for phototoxicity against pancreatic cancer cells.

### 3.2. Physicochemical Characterization of BrCY7-Loaded PEG-PLGA NPs

**Figure 3:**
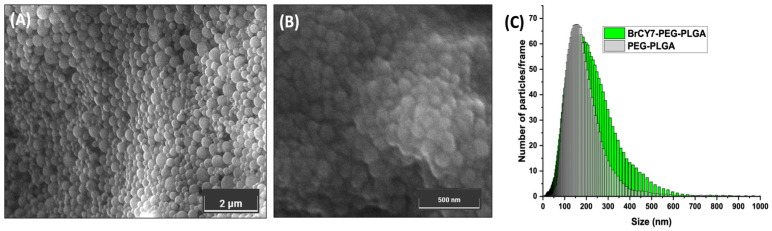
FE-SEM morphology of (**A**) BrCY7-PEG-PLGA and (**B**) empty PEG-PLGA NPs and (**C**) NTA size distribution profile of empty and Br-CY7-loaded PEG-PLGA NPs.

The morphology of both BrCY7-PEG-PLGA NPs and empty PEG-PLGA NPs was observed using FE-SEM (Figure 3A,B). The images reveal a uniform, spherical shape, with an average size of 173 nm. The mean particle diameter (MDP) for both empty and dye-loaded NPs (Figure 3C) was further assessed by nano-tracking analysis (NTA), showing a distinct peak and a relatively narrow, symmetric particle size distribution. Specifically, most of the empty PEG-PLGA NPs are centered around 160 nm and a SPAN value of 1.1, while an increase of approximately 20 nm can be observed for the BrCY7-PEG-PLGA, with a SPAN value of 1.3. These peaks as well as the SPAN values close to 0 highlight the monodispersity of the NPs, indicating that a significant proportion of both empty and BrCY7-PEG-PLGA nanoparticles exhibited a range of size between 160 and 200 nm, which is in line with currently used nanoparticles for drug delivery [41,42]. The slight increase in the calculated size compared to the FE-SEM is reasonable, considering that NTA measures hydrodynamic size, which tends to be larger than the actual size determined by FE-SEM. The obtained ζ-potential values (−24 mV for empty PEG-PLGA and −32 mV for BrCY7-PEG-PLGA) indicate sufficient electrostatic repulsion between particles, ensuring colloidal stability and preventing aggregation [43]. The encapsulation efficiency of BrCY7 in BrCY7-PEG-PLGA was around 85%, with a loading content of 3.4 wt. %.

### 3.3. Spectroscopic Characterization of BrCY7-PEG-PLGA NPs and In Vitro Photodegradation Analysis

**Figure 4:**
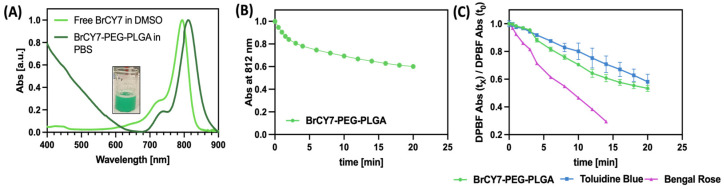
(**A**) Spectroscopic characteristics of the free BrCY7 in DMSO and BrCY7-PEG-PLGA in PBS; (**B**) time-dependent decrease in the DPBF absorption band at 418 nm, monitored under irradiation in the presence of toluidine blue, rose bengal, and BrCY7-loaded PEG-PLGA nanoparticles; (**C**) evaluation of photodegradation of BrCY7 after the incorporation into PEG-PLGA NPs.

As shown in Figure 1B, the absorption peak of free BrCY7 dye dissolved in 100% PBS completely disappears due to the aggregation of this dye in the aqueous environment. Moreover, when dissolved in DMSO and then diluted in PBS, the molecule degrades, exhibiting the appearance of an absorption spectrum to wavelengths of 350 and 450 nm, resulting in molecule breakdown. However, upon incorporation into PEG-PLGA NPs, BrCY7 demonstrated high solubility in aqueous media. In fact, as highlighted in Figure 4A, the absorption spectrum profile of BrCY7-PEG-PLGA in 100% PBS closely resembles the characteristic shape of free BrCY7 when dissolved in DMSO, with a maximum absorption at 812 nm. More importantly, the stability of BrCY7-PEG-PLGA significantly increased, allowing for the investigation of BrCY7-PEG-PLGA ROS production. The increase in absorption at 650 nm is due to the scattering of PEG-PLGA NPs in suspension. In fact, unlike the inset in Figure 1A,B, the sample maintains the typical green color of the molecule, without any evidence of degradation. We finally evaluated the stability of the dye after incorporation into PEG-PLGA NPs and after irradiation. We also evaluated whether the incorporation of the dye into PEG-PLGA nanoparticles could prevent its photodegradation by irradiating the system for different time intervals and measuring the dye absorption at 812 nm. As shown in Figure 4B, a slight photodegradation of BrCY7 can be observed. However, the photodegradation process is relatively slow, allowing for efficient ROS generation, as discussed in the following section. In addition, no appearance of the peak at wavelengths of 350 and 450 nm or change in color were observed for the whole experiment, confirming the protective role of the PEG-PLGA NPs.

### 3.4. ROS Measurement

To preliminarily evaluate the ROS generating ability of BrCY7-PEG-PLGA nanoparticles in PBS and confirm its suitability to be used as PS, the ROS scavenger 1,3-diphenylisobenzofuran (DPBF) was employed, as already reported in the literature [15,35,44], and compared with other widely recognized ROS producer PSs (i.e., toluidine blue and Bengal Rose BR).

As illustrated in Figure 4C, BrCY7-PEG-PLGA nanoparticles exhibit a notable capacity to rapidly generate ROS, outperforming the standard photosensitizer Toluidine Blue. However, their ROS production rate is lower compared to Rose Bengal, likely due to light-scattering effects introduced by the polymeric matrix, which may reduce the effective excitation of BrCY7. It is important to note that the evaluation of the ROS generation assay was conducted in 100% PBS, an environment that did not allow for the solubilization of BrCY7 and led to molecule instability, thus affecting its assessment of ROS production. These results confirmed the suitability of this nanosystem to be used as PS for PDT; thus, the in vitro photoactivity of BrCY7-loaded PEG-PLGA was further investigated against PANC-1 cells.

### 3.5. In Vitro Photoactivity of BrCY7-Loaded PEG-PLGA

The phototoxicity of BrCY7 loaded into PEG-PLGA was investigated on the pancreatic cancer (PANC-1) cell line maintained in physiological (pH_e_ 7.4) medium (PANC-1 CT). Moreover, given the well-established observation that the PDAC microenvironment is more acidic when compared to normal tissues due to a combination of anaerobic or aerobic glycolysis and reduced vascularization that impede the removal of acidic metabolites [45,46], the experiment was also conducted using PANC-1 cells selected for 1 month in pH_e_ 6.6 (PANC-1 pH selected), previously characterized. Indeed, we recently demonstrated that acidic pH selects for a more aggressive PANC-1 phenotype as compared with PANC-1 cells cultured in physiological pH [5].

**Figure 5:**
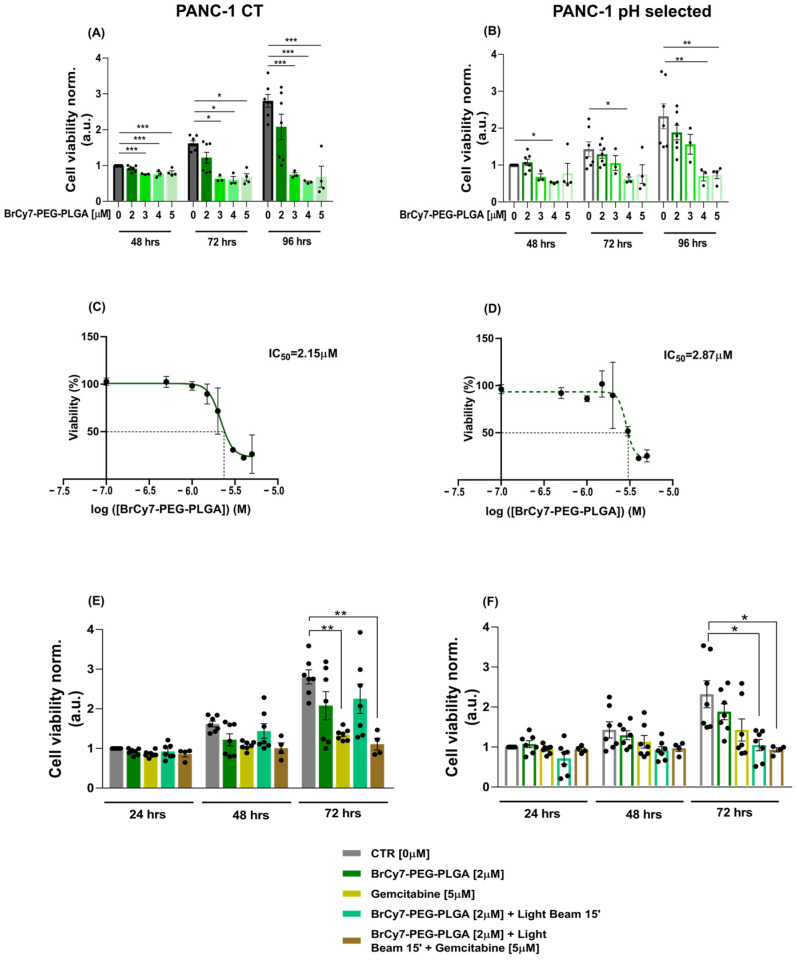
(**A**,**B**) In vitro cytotoxicity of BrCY7-PEG-PLGA in PANC-1 CT cells (**A**) and PANC-1 pH selected cells (**B**). Cell viability assay performed at 48, 72, and 96 h after incubation with 0, 2, 3, 4, and 5 µM of BrCy7-PEG-PLGA. These concentrations refer to the PS concentration, loaded in, respectively, in 0 µg/mL, 500 µg/mL, 750 µg/mL, 1 mg/mL, and 1.250 mg/mL of PEG-PLGA. Data are normalized on CTR 48 h and are represented as the mean (of at least three independent experiments) ± SEM. Statistical significance versus CTR (RM one-way ANOVA without Geisser–Greenhouse correction with Dunnett’s multiple comparisons post hoc test or Friedman with Dunn’s multiple comparisons post hoc test according to data distribution): *: *p*-value < 0.05, **: *p*-value < 0.01, ***: *p*-value < 0.001. (**C**,**D**) IC_50_ curves of BrCY7-PEG-PLGA in PANC-1 CT cells (**C**) and PANC-1 pH selected cells (**D**) 96 h post-treatment. Data are represented as the mean (of at least three independent experiments) ± SEM. (**E**,**F**) In vitro photoactivity of 2 µM BrCY7 loaded in 500 µg/mL PEG-PLGA in PANC-1 CT cells (**E**) and PANC-1 pH selected cells (**F**). Cell viability was evaluated at 24, 48, and 72 h post-irradiation (LB) for 15 min at 770 nm. Gemcitabine [5 µM] was used as the gold standard for the comparison of the BrCY7-PEG-PLGA [2 µM] effect on both cell lines. Data are normalized on CTR (0 µM) at 24 h and represented as the mean (of at least three independent experiments) ± SEM. Statistical significance (RM one-way ANOVA without Geisser–Greenhouse correction with Dunnett’s multiple comparisons post hoc test or Friedman with Dunn’s multiple comparisons post hoc test according to data distribution): *: *p*-value < 0.05, **: *p*-value < 0.01.

We firstly assessed the cytotoxicity of empty PEG-PLGA at different concentrations at varying incubation times (48, 72, and 96 h from nanoparticle incubation) on both PANC-1 CT and PANC-1 pH selected cells. As depicted in Figure S5A,B, PEGylated PLGA nanoparticles exhibit a strong cytocompatibility in both cellular models, in accordance with previous literature [47]. Then, to study the cytocompatibility of the BrCY7 loaded into the nanoparticles, we investigated different concentrations of BrCY7-PEG-PLGA [2 µM, 3 µM, 4 µM, and 5 µM] on both PANC-1 cells’ viability at 48, 72, and 96 h after nanoparticle incubation. Among the different investigated concentrations, in both PANC-1 CT (Figure 5A) and PANC-1 pH selected (Figure 5B), the data clearly show that the presence of BrCy7 causes cytotoxicity in PANC-1 cells at concentrations higher than 2 µM. In fact, the PANC-1 CT cells were already sensitive at 3 µM, 4 µM, and 5 µM after 48 h of incubation; however, PANC-1 pH selected cells were more resistant, as expected, and just the 4 µM and 5 µM resulted in cytotoxicity at all the time points. This is in line with the IC_50_ of the BrCY7-PEG-PLGA, measured in both cellular models. In fact, at 96 h post-treatment with the PS-loaded NPs, the pH selected PANC-1 cells seemed to be slightly more resistant (IC_50_ = 2.87 µM) (Figure 5D) compared to the PANC-1 CT cells, maintained at pH_e_ 7.4 (IC_50_ = 2.15 µM) (Figure 5C). We, therefore, selected the 2 µM concentration of BrCY7-PEG-PLGA for further experiments. The cytotoxicity and phototoxicity of the BrCY7 [2 µM] entrapped in PEG-PLGA [500 µg/mL] were evaluated in both PANC-1 CT (Figure 5E) and pH selected (Figure 5F) cells. Cell viability was assessed at 24, 48, and 72 h after LEDs irradiation. As shown in Figure S6, the light irradiation by itself does not cause an effect on cell viability. The data clearly show that BrCY7-PEG-PLGA does not induce any cytotoxicity nor phototoxicity in PANC-1 CT (Figure 5E) cells. This result can be due to the presence of the polymeric matrix that can scatter the light, reducing the light dose necessary to excite the dye. Interestingly, on the contrary, BrCY7-PEG-PLGA NPs induce a significant phototoxicity effect on pH selected PANC-1 (Figure 5F) cells. The different behavior observed between the two cell lines can be ascribed to the effect of the pH, which is able to swell the polymer matrix, allowing for light penetration and photoexcitation of the dye, as already observed by the authors [35] with similar polymeric nanoparticles.

These data are particularly interesting, considering the high chemotherapeutic resistance developed by PANC-1 cells growing in an acidic microenvironment. Indeed, we compared our nanosystem to gemcitabine, a first-line chemotherapy treatment for PDAC. As expected, irrespective of the concentration, PANC-1 pH selected cells showed a higher resistance to gemcitabine compared with PANC-1 CT cells at 72 h post-treatment (Figure S7), whose viability results were significantly reduced by 50% at lower concentrations of the drug [5 µM]. These results confirm the more aggressive phenotype previously described and the resistance reported in the literature [8].

The data reveal, therefore, a selective phototoxic role for BrCY7-PEG-PLGA [2 µM] in the context of an acidic microenvironment, which is mimicking the central core of PDAC that develops a more resistant phenotype for the first-line gemcitabine treatment. In fact, gemcitabine [5 µM] was ineffective at significantly reducing the cell viability of PANC-1 pH selected cells, on which, on the contrary, the BrCy7-PEG-PLGA [2 µM] displayed significant phototoxicity (Figure 5F). Instead, the less resistant PANC-1 CT cells, cultured at pH_e_ 7.4, are more sensitive to gemcitabine [5 µM] as expected.

Further investigations are needed to better understand if changing the protocol could be sufficient to appreciate an effect of the combination of the two therapies against both PANC-1 CT cells, which seem to be more sensitive to gemcitabine (Figure 5E), and pH selected cells, which are more susceptible to the BrCY7-PEG-PLGA photodynamic effect (Figure 5F). In fact, we believe that building up a combination therapy of gemcitabine and BrCY7-PEG-PLGA could be promising for the treatment of PDAC.

## 4. Conclusions

In the present work, we successfully report the synthesis of a new brominated heptamethine-cyanine dye (BrCY7) with absorption in the NIR region (Abs 795 nm in DMSO) and its incorporation into PEGylated PLGA NPs, improving its photochemical properties in aqueous solution. The NIR nanosystem was then investigated as a possible PS for PDT applications against the pancreatic ductal adenocarcinoma (PDAC) cell line (PANC-1). Its phototoxicity was assessed in an acidic pH environment, using PANC-1 cells adapted for 1 month in pH_e_ 6.6 to obtain more aggressive cells by promoting a more invasive and resistant cell phenotype as compared with control PANC-1 cells (pH_e_ 7.4). Empty PEGylated PLGA nanoparticles exhibited strong cytocompatibility in both CT and pH selected PANC-1 cell lines, confirming its usefulness as a nanocarrier for the delivery of PS for PDT.

We demonstrated that PANC-1 cells maintained at physiological pH_e_ showed a greater susceptibility to the PS-loaded PEG-PLGA, which resulted in slightly more toxicity at a lower concentration (IC_50_ 2.15 µM), compared to the PANC-1 pH selected cells (IC_50_ 2.87 µM).

When irradiated, BrCY7-PEG-PLGA [2 µM] showed a negligible phototoxicity against PANC-1 CT cells; on the contrary, a significant reduction in cellular viability was observed against pH selected PANC-1 cells treated with the same concentration of BrCY7-PEG-PLGA, indicating a possible pH-dependent effect of the nanosystem. This behavior can be ascribed to the effect of the pH, which is able to swell the polymer matrix, allowing for light penetration and photoexcitation of the dye.

Finally, we compared the effect of our nanosystem to gemcitabine, a well-known chemotherapy drug used in the treatment of different cancer types. Gemcitabine significantly reduces the cellular viability of the PANC-1 CT cells, both alone and in the presence of BrCY7 [2 µM]-loaded NPs, upon irradiation.

As far as PANC-1 pH selected cells, gemcitabine alone did not significantly reduce cellular viability, confirming the resistance to the treatment, but when treated with the PS-loaded PEG-PLGA and irradiated with LEDs at 770 nm, we could observe a significant reduction in cell viability. These data confirm that BrCY7-PEG-PLGA phototoxicity is more effective than gemcitabine in the treatment of resistant PDAC cell lines, highlighting the potential of BrCY7-PEG-PLGA NPs in PDT for the treatment of chemotherapy-resistant tumors, including PDAC. A deeper understanding of nanoparticle degradation under acidic conditions, particularly within the tumor microenvironment, remains an important aspect to explore. Future studies should focus on a comprehensive stability assessment at different pH levels, examining variations in particle size, surface charge, and potential polymer degradation over time. Additionally, translating these findings to in vivo applications presents inherent challenges, including potential differences in nanoparticle behavior, biodistribution, and therapeutic efficacy. Addressing these aspects would provide valuable insights into their behavior in biological environments and enhance their potential for controlled drug release.

## Data Availability

The original contributions presented in this study are included in the article/Supplementary Materials. Further inquiries can be directed to the corresponding author.

## Supplementary Materials

The following supporting information can be downloaded at: https://www.mdpi.com/article/10.3390/polym17081101/s1, Figure S1. ^1^H NMR of BrCY7 in CDCl_3_. Figure S2. ^13^C NMR of BrCY7 in CDCl_3_. Figure S3. HR-MS of BrCY7. Figure S4. Evaluation of molar extinction coefficient. Absorbance intensities of each BrCY7 solution at the *λ*_max_ were plotted versus the sample concentration. A linear fit was applied to determine the molar extinction coefficient (*ε*) as the slope of the line. Figure S5. (A,B) in vitro cytotoxicity of PEG-PLGA nanocarrier in PANC-1 CT (A) and PANC-1 pH selected (B). Cell viability assay performed at 48, 72 and 96 h after incubation with 0 µg/mL (CTR), 500 µg/mL, 750 µg/mL, 1 mg/mL and 1.250 mg/mL of PEG-PLGA. Data are normalized on CTR 48 h and are represented as mean (of at least three independent experiments) ± SEM. Statistical significance versus CTR (RM one-way ANOVA without Geisser-Greenhouse correction with Dunnett’s multiple comparisons post-hoc test or Friedman with Dunn’s multiple comparisons post-hoc test according to data distribution). Figure S6. Effect of Light irradiation on untreated cells, both PANC-1 CT (filled) and PANC-1 pH selected (empty). Cell viability assay performed at 24, 48 and 72 h post adhesion of cells. Data are normalized on CTR 24 h and are represented as mean (of seven independent experiments) ± SEM. Statistical significance of CTR + Light Beam 15’ versus CTR has been for all the conditions using Parametric *t*-Test. Figure S7. Gemcitabine dose-response curve of PANC-1 CT (continuous black line) and PANC-1 pH selected (dashed black line). Cells have been treated with gemcitabine 5, 10, 50 µM and the cell viability has been measured at 72 h post treatment. Data are normalized on untreated cells (0 µM) and are represented as mean (of three independent experiments) ± SEM. Statistical significance versus untreated cells (RM one-way ANOVA without Geisser-Greenhouse correction with Dunnett’s multiple comparisons post-hoc test or Friedman with Dunn’s multiple comparisons post-hoc test according to data distribution): **: *p*-value < 0.01, ***: *p*-value < 0.001 (PANC-1 CT); #: *p* < 0.05, ##: *p* < 0.01 (for PANC-1 pH selected).
