# Supplementary material for: NIR pH-Responsive PEGylated PLGA Nanoparticles as Effective Phototoxic Agents in Resistant PDAC Cells
Source: Polymers (Basel). 2025 Apr 18;17(8):1101. doi: 10.3390/polym17081101 (PMC12030558; doi:10.3390/polym17081101)
Supplement: Supplementary file 1 [file polymers-17-01101-s001.zip › polymers-3575606-supplementary.pdf]

Supplementary information

NIR pH-responsive PEGylated PLGA nanoparticles as effective phototoxic agents in **resistant PDAC cells**

Degnet Melese Dereje[1,2,†], Francesca Bianco[3,4,†], Carlotta Pontremoli[1*], Alessandra Fiorio Pla[3], Nadia Barbero[1,5]

[1]Department of Chemistry, NIS Interdepartmental and INSTM Reference Centre, University of Torino, Via G. Quarello 15A, 10135 Torino, Italy.

[2]Department of Chemical Engineering, Bahir Dar Institute of Technology, Bahir Dar University, Polypeda 01, Bahir Dar 0026, Ethiopia.

[3]University of Torino, Department of Life Sciences and Systems Biology, Via Accademia Albertina 13, 10123 Turin, Italy.

[4]University of Perugia, Department of Chemistry, Biology and Biotechnology, Via dell' Elce di Sotto 8, 06123, Perugia, Italy.

[5]Institute of Science and Technology for Ceramics (ISSMC-CNR), Via Granarolo, 64, 48018 Faenza, Italy.

*Correspondence: carlotta.pontremoli@unito.it

† These authors contributed equally to this paper

Supplementary information

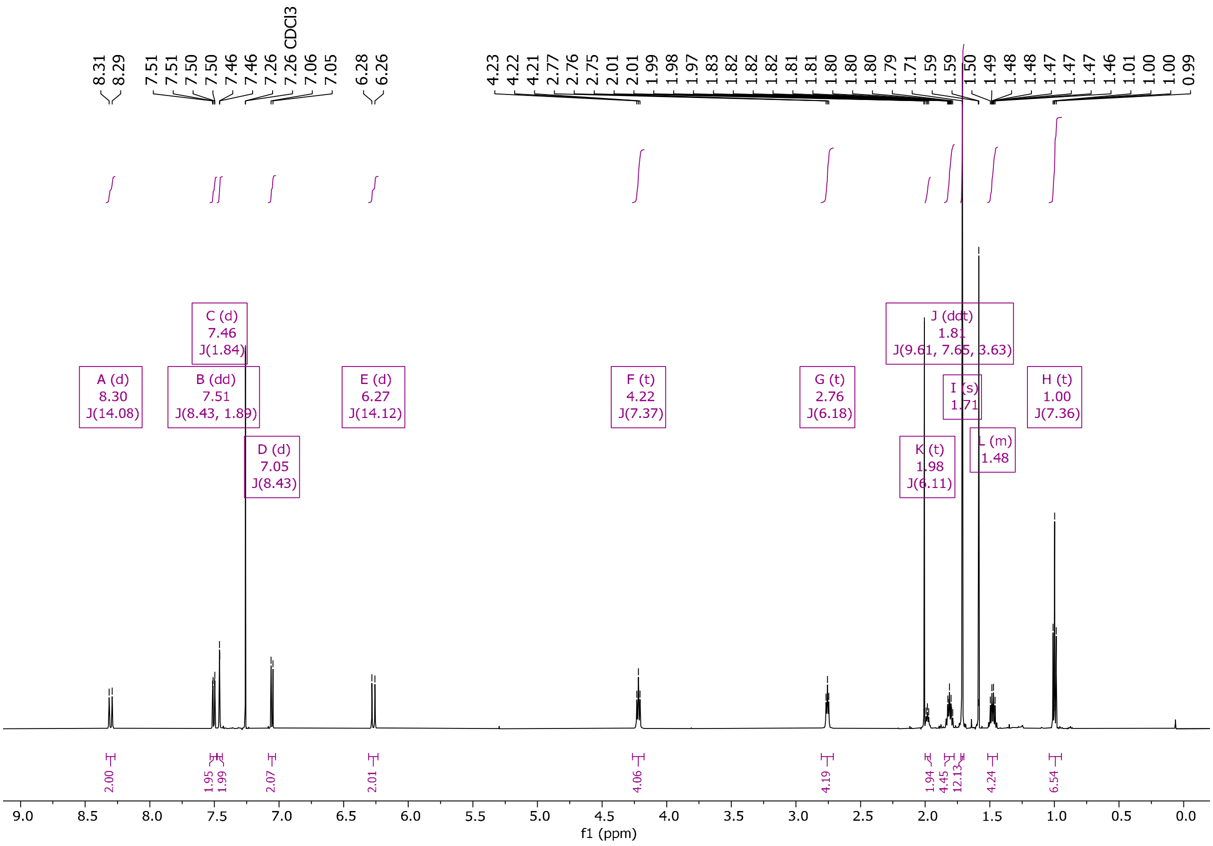

Figure S1. ^{1}H NMR of **BrCY7** in $CDCl_3$.

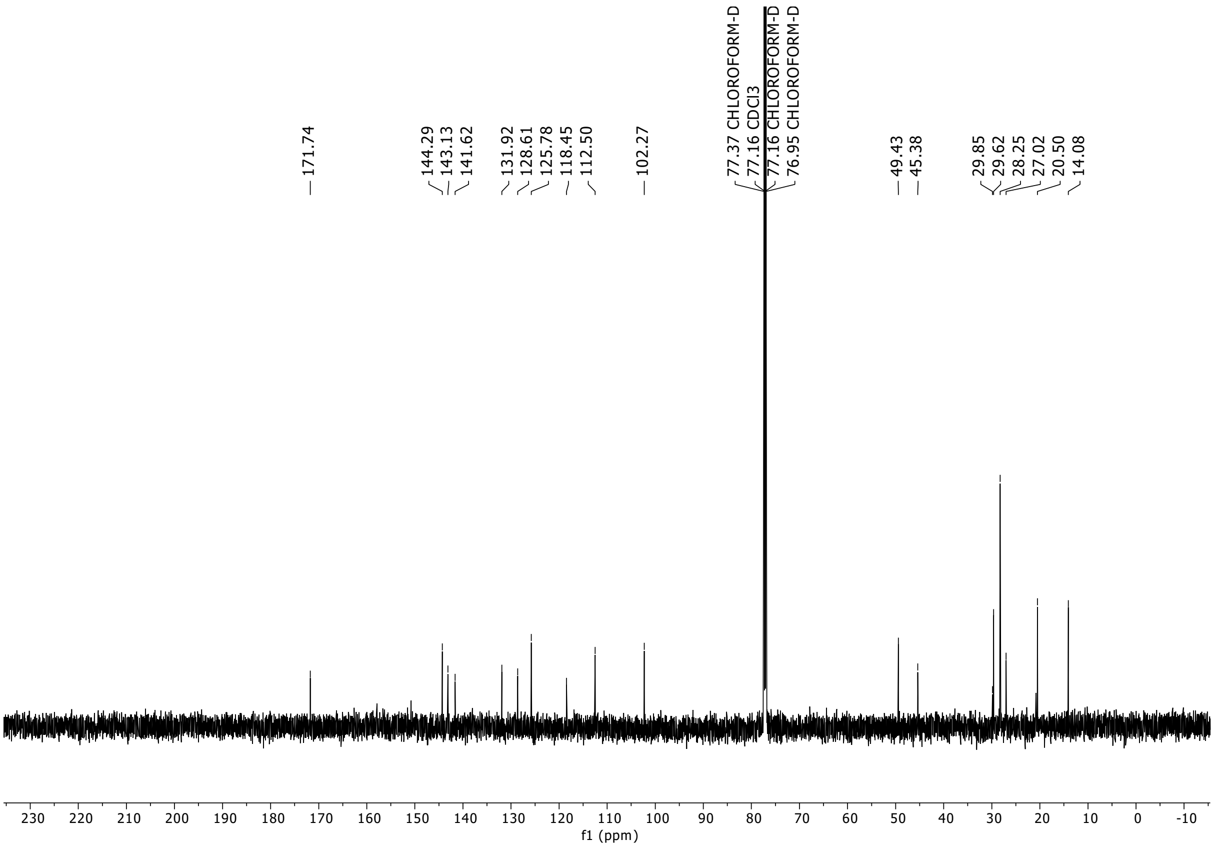

Figure S2. ^{13}C NMR of **BrCY7** in $CDCl_3$.

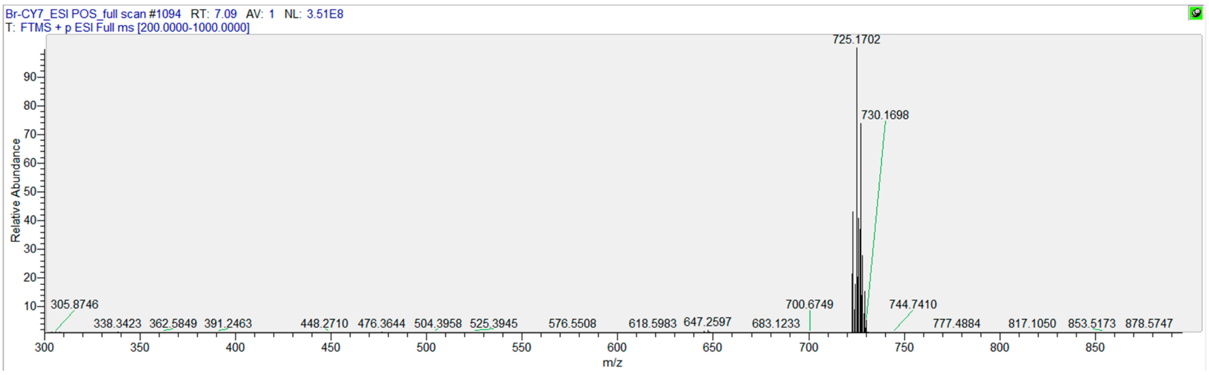

Figure S3. HR-MS of **BrCY7**.

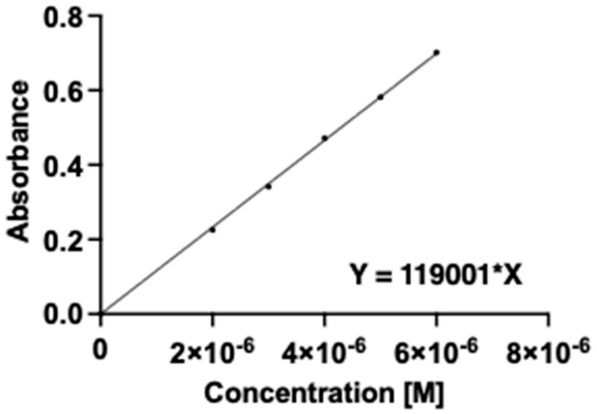

Figure S4. Evaluation of molar extinction coefficient. Absorbance intensities of each BrCY7 solution at the λ_{max} were plotted versus the sample concentration. A linear fit was applied to determine the molar extinction coefficient (ε) as the slope of the line.

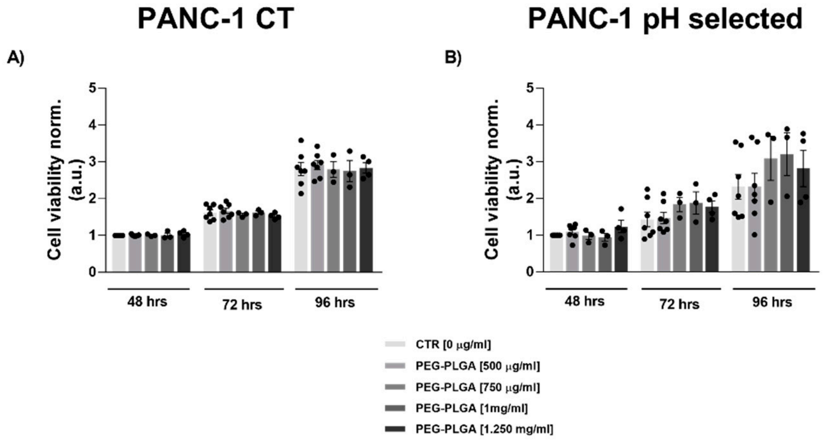

Figure S5. A-B) *in vitro* cytotoxicity of PEG-PLGA nanocarrier in PANC-1 CT (A) and PANC-1 pH selected (B). Cell viability assay performed at 48, 72 and 96 hours after incubation with 0 µg/mL (CTR), 500 µg/mL, 750 µg/mL, 1 mg/mL and 1.250 mg/mL of PEG-PLGA. Data are normalized on CTR 48 hr and are represented as mean (of at least three independent experiments) ± SEM. Statistical significance versus CTR (RM one-way ANOVA without Geisser-Greenhouse correction with Dunnett's multiple comparisons post-hoc test or Friedman with Dunn's multiple comparisons post-hoc test according to data distribution).

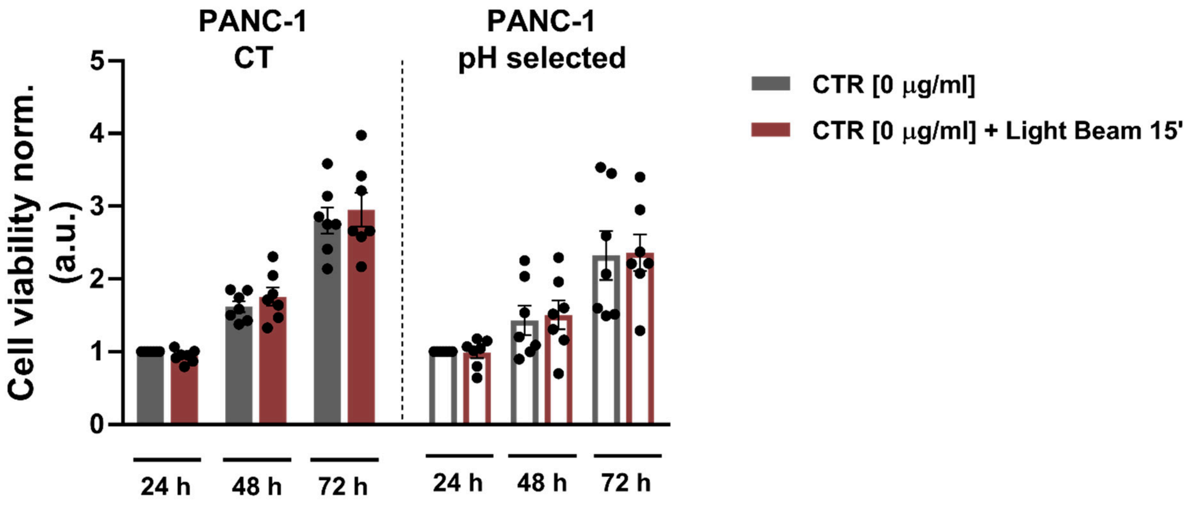

Figure S6. Effect of Light irradiation on untreated cells, both PANC-1 CT (filled) and PANC-1 pH selected (empty). Cell viability assay performed at 24, 48 and 72 hours post adhesion of cells. Data are normalized on CTR 24 hr and are represented as mean (of seven independent experiments) ± SEM. Statistical significance of CTR + Light Beam 15’ versus CTR has been for all the conditions using Parametric T-Test.

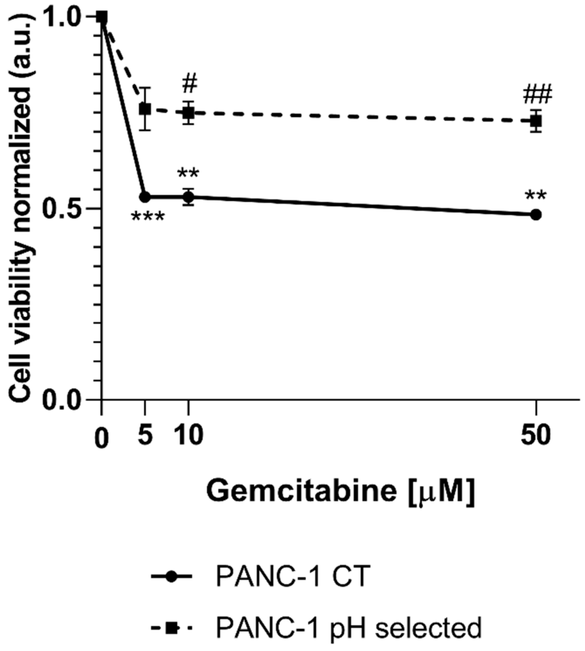

Figure S7. Gemcitabine dose- response curve of PANC-1 CT (continuous black line) and PANC-1 pH selected (dashed black line). Cells have been treated with gemcitabine 5, 10, 50 μM and the cell viability has been measured at 72 hours post treatment. Data are normalized on untreated cells (0 μM)

and are represented as mean (of three independent experiments) ± SEM. Statistical significance versus untreated cells (RM one-way ANOVA without Geisser-Greenhouse correction with Dunnett's multiple comparisons post-hoc test or Friedman with Dunn's multiple comparisons post-hoc test according to data distribution): **: p-value < 0.01, ***: p-value<0.001 (PANC-1 CT); #: $p<0.05$, ##: $p<0.01$ (for PANC-1 pH selected).
